# What matters to patients? A timely question for value-based care

**DOI:** 10.1371/journal.pone.0227845

**Published:** 2020-07-09

**Authors:** Meron Hirpa, Tinsay Woreta, Hilena Addis, Sosena Kebede

**Affiliations:** 1 Department of Medicine, Johns Hopkins School of Medicine, Baltimore, Maryland, United States of America; 2 Division of Gastroenterology and Hepatology, Johns Hopkins University School of Medicine, Baltimore, Maryland, United States of America; 3 Clinical Research Unit, University of Pennsylvania, Philadelphia, Pennsylvania, United States of America; 4 Johns Hopkins Community Physicians – Remington, Baltimore, Maryland, United States of America; Massachusetts General Hospital, UNITED STATES

## Abstract

**Background:**

Our healthcare system is moving towards patient-centered and value-based care models that prioritize health outcomes that matter to patients. However, little is known about what aspects of care patients would prioritize when presented with choices of desirable attributes and whether these patient priorities differ based on certain demographics.

**Objective:**

To assess patients’ priorities for a range of attributes in ambulatory care consultations across five key health service delivery domains and determine potential associations between patient priorities and certain demographic profiles.

**Methods:**

Using a *What Matters to You* survey patients ranked in order of importance various choices related to five health service domains (patient-physician relationship, personal responsibility, test/procedures, medications, and cost). Subjects were selected from two Johns Hopkins affiliated primary care clinics and a third gastroenterology subspecialty clinic over a period of 11 months. We calculated the percentage of respondents who selected each quality as their top 1–3 choice. Univariate and multivariate analyses determined demographic characteristics associated with patient priorities.

**Results:**

Humanistic qualities of physicians, leading a healthy lifestyle, shared decision making (SDM) for medications and tests/procedures as well as knowledge about insurance coverage were the most frequently ranked choices. Privately insured and more educated patients were less likely to rank humanistic qualities highly. Those with younger age, higher educational attainment and private insurance had higher odds of ranking healthy lifestyle as a top choice. Those with more education had higher odds of ranking SDM as a top choice.

**Conclusions:**

Identifying what matters most to patients is useful as we move towards patient-centered and Value Based Care Models. Our findings suggest that patients have priorities on qualities they value across key health service domains. Multiple factors including patient demographics can be predictors of these priorities. Elucidating these preferences is a challenging but a valuable step in the right direction.

## Introduction

A growing body of literature advocates for Patient Centered Care (PCC) which is defined as “care that is respectful of, and responsive to, individual patient preferences, needs and values, and ensuring that patient values guide all clinical decisions.” [1] Many argue that focusing health care around the needs and preferences of patients has the potential to improve clinical outcomes, quality of care and patient satisfaction while decreasing healthcare costs and health disparities. [2–7] Thus, another way of looking at the core principle behind patient centeredness is the intentional alignment of health service delivery with what matters to patients most.

The healthcare system is also trying to find ways to move away from a fee-for-service model that is organized around the volume of care to a fee-for-value model. One commonly accepted definition of value-based care (VBC) is “the creation and operation of a health system that explicitly prioritizes health outcomes which matter to patients relative to the cost of achieving this outcome.” [8] The Centers for Medicare & Medicaid Services which has recently implemented a value-based purchasing (VBP) program that ties reimbursement to quality or value identifies “Person and Community Engagement” as one of the four key domains of VBP (the other three domains are “Safety”, “Clinical Care” and “Efficiency and Cost Reduction”). [9] Several health systems are also beginning to utilize similar measures that incorporate patient experience into payment models, reflecting the broader movement towards PCC which places the patient at the center of care delivery. [10] It is clear that an alignment between PCC and VBC will require the explicit and purposeful integration of patient perspectives and preferences into quality metrics, guidelines and clinical decisions that influence the delivery of care. [11] Without this alignment and focus on patient preferences, it will not be possible to achieve the objective of VBC which aims to achieve higher-quality health services and cost containment.

One challenge in the measurement of patient experience is the difficulty of differentiating among multiple overlapping terms like satisfaction, engagement, perceptions, priorities, values and preferences. [12–15] Patient preference and value can also be highly dynamic and dependent on several factors including patients’ health status, and personal characteristics such as education level. [16–19]

Despite the limitations of patient-reported measures, patient surveys can provide helpful data to identify patient preferences and values. Patients are often more satisfied with health care services that are delivered to meet their preferences. [10,20] Patients have also been known to value Shared Decision Making (SDM), a process where health care providers involve patients more actively as partners in decision making, incorporating both medical evidence and individual patient priorities and preferences. [21,22] Thus, knowledge about patients’ priorities can increase value for patients by improving patient satisfaction, the delivery of patient-centered health services, quality of care and outcomes. [12,16,23]

Patients value both the *clinical* (quality of clinical care: such as provider knowledge and skill) and the *interpersonal* (quality of communication: such as SDM) qualities of care. [24–26] Multiple studies identify patient-provider communication, humanistic qualities and SDM to be the most important aspects of care that patients value for high-quality health care regardless of variations in socio-demographic or health characteristics of patients. [10,17,21,22,25,27–29] Some evidence suggests that when choosing a primary care physician, the majority of patients have a strong preference for physicians of high clinical quality if forced to make a tradeoff between interpersonal and clinical skills. [19,30–32]

How value of the various attributes of healthcare may vary by certain patient demographics and reasons for presentation in the ambulatory primary care setting has been postulated before. [15,33] There is data that suggests low-income patients, those with a high prevalence of psychosocial problems and those feeling unwell have a preference for good communication and personal interaction when compared to their counterparts. [3,15,34] Prior studies also show that differences in race, education level and socio-economic status lead to differences in patients’ health care-related beliefs, practices, and values. [35–37] A study by Levinson et al. showed that women and more educated people were more likely to prefer an active role in decision making while African-Americans and Hispanics preferred that physicians make decisions. [38] Some studies have also shown that older patients are less likely to prioritize good communication and SDM [3,19,38] whereas other studies show that older patients at the end of life valued effective communication and trust of the provider. [34] Other studies have also shown that highly educated patients and those from a higher socioeconomic status are more likely to have a preference for healthy lifestyles. [39–42]

While prior studies help identify several aspects of care that patients identify as important, resource scarcities limit the feasibility of implementing all aspects of care that are important to patients. Thus, it is valuable to identify which aspects of care are most important to patients. It is also important to identify differences in preferences along particular patient groups/demographics. There is limited research examining how patients would prioritize a list of desirable attributes about specific aspects of their care, if forced to make choices. To our knowledge, no study has examined patients’ priorities across key healthcare domains that we tested with concurrent assessment of demographic associations. Knowledge about specific patient priorities and demographic associations can define value from health outcomes that matter to specific groups of patients and in turn allow for a more targeted approach in designing and implementing a VBC model of service delivery.

Though several health institutions are beginning to incorporate questions about what matters to patients into their patient intake forms with improved reported patient satisfaction [43], the question is enormously layered and there is no validated survey for its application. To get a more granular answer as to what matters to patients in different health care categories, we designed a survey with 5 domains through which patients routinely experience their healthcare providers in the outpatient setting: patient- physician relationship, patient’s personal health responsibility, tests/procedures, medications and cost of care. We selected these 5 categories since they have been identified as key components of patient-satisfaction in multiple healthcare related studies (albeit not in combination). [25,44–47] For instance, patient-physician interaction is noted to have one of the strongest impacts on patient satisfaction and that along with medications is one of the composite questions found in the validated tool Consumer Assessment of Healthcare Providers and Systems (CAHPS). [48] The other reason we picked these 5 categories was because three out of these five domains were in fact the subject of an inpatient study we did in 2014 at the Johns Hopkins Hospital [44]. The objective of that study was to find out the level of concordance between discharged patients’ understanding of their diagnoses, medications and procedures/tests and their physicians’ documentation (“Shared Understanding”) and if the level of concordance determined patient satisfaction. To our current outpatient study, we added 2 additional domains- “personal health responsibility” and “cost”. These two domains are more relevant for outpatient primary care interaction because the current trend towards VBC emphasizes preventive measures and cost containment.

This study assessed patients’ priorities for a range of attributes in ambulatory care consultations across the above listed 5 domains (patient-physician relationship, personal responsibility, tests/procedures, medications and healthcare cost) and then examined potential association between patient priorities with certain demographic profiles. Our main outcome measures were based on the participants’ ranking of three to four important qualities under each of the five domains in the order of their personal priority.

There are 3 specific outcome measures we looked at:

1. What specific qualities under each healthcare domain were most frequently ranked as the number one choice.
2. What patients ranked as their second and third choices.
3. Patients’ demographics as potential predictors of the most frequent top choice under each of the five healthcare domains.

## Methods

### Design

This study was a paper-based, self-administered survey in English designed to assess patient preferences surrounding the healthcare service they receive. The survey asked patients to rank in order of importance various choices related to the 5 domains (see Table 2 and S1 Appendix). On Question 1 (patient-physician relationships) patients were asked to rank 7 choices in order of importance from 1 (most important) to 7 (least important). On Question 2 (patients’ personal responsibility on health) patients were asked to rank 3 choices in order of importance from 1(most important) to 3 (least important). On Question 3 (diagnostic tests) patients were asked to rank 3 choices in order of importance from 1(most important) to 3 (least important). On Question 4 (patient preferences regarding medications) patients were asked to rank 5 choices in order of importance from 1(most important) to 5 (least important). On Question 5 (healthcare costs) patients were asked to rank 3 choices in order of importance from 1(most important) to 3 (least important).

To determine whether priorities varied among subgroups of patients, we collected demographic data including age, sex, ethnicity, highest level of education and type of medical insurance.

### Set up and study population

This study was conducted in Baltimore, Maryland, a city with a population of approximately 593,490 people. [49] The subjects of this study were patients being evaluated at two Johns Hopkins affiliated primary care clinics and a third gastroenterology subspecialty clinic: These clinics operate under the Johns Hopkins Community Physicians (JHCP) network that takes care of approximately 900,250 patients each year in its more than 40 outpatient clinics. [50] At the three clinic sites, an average of 6–10 patients are seen in a half-day clinic session per provider. The Johns Hopkins Institutional Review Board and the Johns Hopkins Community Physicians Research and Projects Committee approved this study. All participants were advised, verbally and in a written form, that their completion of the survey will serve as their consent to be in the research study.

### Survey development

The first step in the development of the study was to select 5 domains through which patients routinely experience their healthcare providers as described in the introduction.

The second step was to consider the use of rating versus ranking scales. Since our aim was to identify if patients had priorities among a range of desirable attributes in a select set of healthcare domains, a ranking scale was appropriate. Our scales ranged 3–7 categories under each domain, which is consistent among most similarly designed surveys. [51]

We then piloted the survey using 23 randomly selected patients at the JHCP at Remington location. We found out that the most common problem with the initial survey was patients tended to rate different categories equally despite instructions to prioritize. Based on this we adjusted our survey scales and clarified instructions. In order to improve the reliability of our survey, we avoided jargons and complex words.

The results from the pilot survey were similar to our final finding in that most patients rated humanistic qualities of physicians highest giving us a measure of confidence that our final survey has a reasonable reliability for our patient population.

### Survey administration

From 7/1/2018-6/30/2019, a total of 338 consecutive patients were surveyed prior to seeing their physician in clinic. A predominant number of patients surveyed (n = 298) were primary care patients. After patients were roomed for their visit, before seeing their physicians, all patients were asked by either a nurse or a physician if they would participate in a 5-10-minutes self-administered paper-based survey in English. The number of patients who received surveys were variable in a clinic session based on show rates. On average the response rate was above 90%. Please see survey attached in S1 Appendix.

### Data analysis

A total of 338 patients were surveyed in this study. Incomplete and erroneously completed questionnaires (n = 112) were excluded from analysis. Data from the accurately completed questionnaires (n = 226, 196 of which were primary care patients) were aggregated and analyzed using Excel and Stata 15.1. Some patients inadvertently received a version of the survey that had 4 choices for question four instead of 5 choices and 4 choices for question 5 instead of 3 choices. Therefore, of the accurately completed surveys (n = 226), an additional 53 and 95 surveys were excluded from analysis for questions 4 and 5, respectively. As a result, when calculating percent respondents for questions 4 and 5, 173 surveys for question 4 and 132 surveys for question five were analyzed.

To assess which qualities were most important for patients under each of the five domains, the percentage of respondents who selected each quality as their number one choice were determined. Since patients were forced to prioritize among a list of desirable attributes, the qualities that were ranked as the most frequent second and third choice were also determined. For questions that had greater than or equal to four choices (Questions 1 and 4), the most frequent first, second, and third choices were calculated while only the most frequent first and second choices were calculated for questions that had three choices (Questions 2, 3 and 5).

Univariate and multivariate logistic regression analyses were used to determine whether patient characteristics such as age, sex, race, education, and insurance type were significant predictors of the choosing the qualities most frequently ranked as number one for each of the five domains.

For each domain, we determined the quality that was most frequently selected as the number one priority by participants. We then looked at the binary outcome of whether a participant selected this quality as their number one priority or not. We performed univariate logistic analysis to explore the association of these patient characteristics with whether they selected this quality as their number one priority. We created a final multivariate model incorporating all of these patient characteristics as independent variables as we deemed each of them to be important determinants of patients’ prioritization of qualities.

During analysis, for Question 1, the choices “Kindness” and “Efforts to connect with me as a human being and not just as a patient” were combined under the heading “humanistic qualities”. For Question 2, survey option “Learn as much as I can about my condition and be actively involved in decision making” was categorized as “SDM”. For Question 4, survey options “I want to know exactly what I am taking and why” and “I want to understand the side effects of each medication thoroughly before accepting the prescription” were combined under the heading “SDM”.

## Results

Our main results showed that humanistic qualities of physicians (for Q 1), leading a healthy lifestyle (for Q 2), shared decision making (SDM) for medications and tests/procedures (for Q3 and for Q4) and knowledge about insurance coverage (for Q5) were the most frequently ranked top choices. Privately insured and more educated patients were less likely to rank humanistic qualities highly. Those with younger age, higher educational attainment and private insurance had higher odds of ranking healthy lifestyle as a top choice. Those with more education had higher odds of ranking SDM as a top choice.

Table 1 shows the distribution of participants according to age, gender, race, education level and health insurance. The participants are mainly middle aged (mean age 42.6 years), female (77.9%), college educated (54%) and privately insured (74.1%). There were about an equal percentage of Blacks (41.6%) and Whites (44.7%).

**Table 1:** Demographics of overall study population (N = 226).

Characteristic	N (%)
Age (years)	42.6* (19–83)
Sex	
Female	176 (77.9)
Male	50 (22.1)
Race	
Black	94 (41.6)
White	101 (44.7)
Other	31 (13.7)
Education	
High school or less	41 (18.1)
Some college	63 (27.9)
College graduate	32 (14.2)
Postgraduate degree	90 (39.8)
Insurance	
Medicaid	24 (10.9)
Medicare	27 (12.3)
Private	163 (74.1)
Other	6 (2.7)

*Mean (range)

Table 2 shows the percentage of patient respondents who ranked each quality as number one under each of the five domains. Participants chose humanistic qualities of physicians, leading a healthy lifestyle, SDM for medications and tests/procedures and knowledge about insurance coverage as their top choices. Specifically, for question one assessing patient-physician relationship, “humanistic qualities” (33%) was the most frequent number one choice while knowledge of the physician and ability to explain things fully were tied at 23% as the second most frequent top choice. For question number two assessing patient personal responsibility, leading a healthy lifestyle (47%) was the most frequent top choice while SDM (35%) and following medical recommendations (18%) were the second and third top choices, respectively. For question number three on tests and procedures, the most frequent top choice was SDM (50%) while wanting all tests that could be helpful (43%) and only wanting the absolute critical tests (7%) were the second and third top choices, respectively. On question four assessing medications, SDM (80%) was the most frequent top choice while wanting the absolute minimum medications (9%) and wanting any medication that could help (9%) were the most frequent second choice. Wanting the freedom to try alternative medicine and herbal supplements (2%) was the least frequent choice. On question five assessing healthcare cost, knowing what insurance covers (57%) was the most frequent choice while knowing what charges are for (32%) and minimizing healthcare expenditure (11%) were the second and third choices, respectively.

**Table 2:** The percentage of respondents who selected each quality as top choice.

**Patient-Physician Relationship N = 226**	**Percent Respondents**
Humanistic qualities^1^	33%
Fund of knowledge	23%
Explaining things fully and in the way I understand	23%
Involving me in decision-making	8%
Being on time	7%
Spending adequate time with me	6%
**Personal Responsibility N = 226**	**Percent Respondents**
Exercise, diet and lead a healthy lifestyle	47%
Shared decision making (SDM)^2^	35%
Follow medical recommendations given	18%
**Tests and Procedures N = 226**	**Percent Respondents**
Shared decision making (SDM)	50%
I want all the tests that could be helpful to understand my condition better	43%
I only want the absolute critical tests to be performed	7%
Medications N = 173*	**Percent Respondents**
Shared decision making (SDM)^3^	80%
I want the absolute minimum that I need to take for my condition	9%
I want to take anything that can possibly help my condition	9%
I want the freedom to try alternative medicine and herbal supplements	2%
Healthcare Cost N = 132**	**Percent Respondents**
I want to know what my health insurance covers	57%
I want to know exactly what I am being charged for	32%
I want to minimize my healthcare expenditure	11%

^1^ Combines respondents who picked the survey options “Kindness” and “Efforts to connect with me as a human being and not just as a patient”.

^2^ Survey option was “Learn as much as I can about my condition and be actively involved in decision making”.

^3^ Combines respondents who picked the survey options “I want to know exactly what I am taking and why” and “I want to understand the side effects of each medication thoroughly before accepting the prescription”.

*Excluding 53 participants who were provided 4 choices instead of 5 choices for Question #4

**Excluding 95 participants who were provided 4 choices instead of 3 choices for Question #5

Table 3 shows the top two or three most frequently selected qualities for each question. Recall that in Table 2 we reported only the top qualities selected for each domain. Here we are reporting the second and third top qualities, in addition to the top choice selected for each question. Supporting the findings in Table 2, humanistic qualities of physicians was reported as not only the primary choice but also as the 2^nd^ and 3^rd^ most frequent choice. Similarly, SDM (understanding importance of diagnostic tests and understanding indication and side effects of medications) was reported as the most frequent 1^st^ and 2^nd^ choice when it came to diagnostic tests and medications. However, for the Personal Responsibility and Cost domains, respondents reported a different secondary choice. Specifically, for question one assessing patient-physician relationship, humanistic qualities was the most frequent first (33%), second (24%) and third (30%) choice. For question number two assessing personal responsibility, healthy lifestyle (47%) was the most frequent top choice while learning about condition (38%) was the most frequent second choice. For tests and procedures, understanding the importance of diagnostic tests was the most frequent first (50%) and second (39%) choice. On question four assessing medications, understanding indication and side effects of medications was the most frequent first (80%), second (57%) and third (41%) choice. For question five on healthcare cost, knowing what insurance covers (58%) was the most frequent first choice while understanding charges (43%) was the most frequent second choice.

**Table 3:** Top 1–3 qualities selected by respondents^4^.

Questions	Most frequent first choice	Most frequent second choice	Most frequent third choice
Q 1 (Patient-physician Relationship)	Humanistic Qualities	Humanistic Qualities	Humanistic Qualities
N = 226	**33%**	**24%**	**30%**
Q 2 (Personal Responsibility)	Healthy Lifestyle	Learning about condition	____
N = 226	**47%**	**38%**	
Q 3 (Tests and Procedures)	Understand importance of diagnostic tests	Understand importance of diagnostic test	____
N = 226	**50%**	**39%**	
Q 4 (Medications)	Understand indication and side effects of medications	Understand indication and side effects of medications	Understand indication and side effects of medications
N = 173*	**80%**	**57%**	**41%**
Q 5 (Healthcare cost)	Know what insurance covers	Know what my charges are	____
N = 132**	**58%**	**43%**	

^4^ For questions that had four or more choices (Questions 1 and 4), the most frequent first, second, and third choices were calculated while the most frequent first and second choices was calculated for questions that had three choices (Questions 2, 3 and 5).

*Excluding 53 participants who were provided 4 choices instead of 5 choices for Question #4

**Excluding 95 participants who were provided 4 choices instead of 3 choices for Question #5

Table 4 shows univariate analysis for demographic predictors of the most frequent top choice for each question (Q1: “humanistic qualities”; Q2: “healthy lifestyle”; Q3: “SDM”; Q4: “SDM” and Q5: “knowing insurance coverage”). Age (“older”), race (“other”), level of education (“college or above”), type of insurance (“private”) seem to affect preferences of respondents. Specifically, when assessing patient-physician relationship, patients with college and above degrees and those with private insurance were less likely to rank humanistic qualities as their top choice compared to their references (0.55, CI 0.31–0.98 and 0.26, CI 0.11–0.64, respectively). For question two assessing patient personal responsibility, those 45 and older were less likely to rank healthy lifestyle as their number one choice when compared to those younger than 35 (0.20, CI 0.10–0.41 and 0.29, CI 0.11–0.77). Participants who identified their race as “Other”, those who had a college and above education and privately insured patients had higher odds of ranking healthy lifestyle as their number one choice compared to their references (2.55, CI 1.11–5.87; 4.25, CI 2.28–7.91 and 8.42, CI 2.42–29.33, respectively). When assessing preferences on tests and procedures, SDM was less likely to be ranked as a number one choice by those older than 65 (0.35, CI 0.13–0.99) but more likely to be ranked as a top choice by those with college and above education (2.01, CI 1.14–3.55) when compared to their respective references. With regards to medications, those who identified their race as “Other” had lower odds of choosing SDM as their top choice when compared to Blacks (0.24, CI 0.08–0.71). We saw no significant predicators for question five that assessed healthcare cost.

**Table 4:** Univariate analysis for predictors of most frequent top choice for each question.

	Question #1 Patient-physician Relationship	Question #2 Personal Responsibility	Question #3 Tests and Procedures	Question #4 Medications	Question #5 Healthcare cost
Most frequent top choice	Humanistic Qualities	Healthy Lifestyle	Shared Decision Making (SDM)	Shared Decision Making^3^	Know Insurance coverage
Age (in years)	OR (95% C.I.)	OR (95% C.I.)	OR (95% C.I.)	OR (95% C.I.)	OR (95% C.I.)
< 35	1 [Reference]	1 [Reference]	1 [Reference]	1 [Reference]	1 [Reference]
35–44	0.80 (0.39–1.64)	0.53 (0.27–1.05)	1.48 (0.75–2.93)	0.44 (0.15–1.26)	1.04 (0.42–2.58)
45–64	0.69 (0.34–1.41)	**0.20 (0.10–0.41**)	0.97 (0.50–1.87)	0.38 (0.14–1.03)	1.08 (0.45–2.60)
> = 65	1.11 (0.43–2.88)	**0.29 (0.11–0.77)**	**0.35 (0.13–0.99)**	0.28 (0.07–1.16)	0.88 (0.27–2.83)
Sex					
Female	1 [Reference]	1 [Reference]	1 [Reference]	1 [Reference]	1 [Reference]
Male	0.83 (0.42–1.64)	0.68 (0.36–1.29)	0.59 (0.31–1.13)	0.61 (0.26–1.41)	0.57 (0.25–1.30)
Race					
Black	1 [Reference]	1 [Reference]	1 [Reference]	1 [Reference]	1 [Reference]
White	0.90 (0.50–1.62)	1.71 (0.97–3.02)	1.55 (0.88–2.72)	0.63 (0.26–1.53)	0.65 (0.30–1.38)
Other	0.76 (0.31–1.83)	**2.55 (1.11–5.87)**	1.57 (0.69–3.55)	**0.24 (0.08–0.71)**	0.31 (0.10–1.04)
Education					
Some college	1 [Reference]	1 [Reference]	1 [Reference]	1 [Reference]	1 [Reference]
Or below	**0.55 (0.31–0.98)**	**4.25 (2.28–7.91)**	**2.01 (1.14–3.55)**	1.03 (0.46–2.30)	
College and					0.51 (0.25–1.06)
Above					
Insurance					
Medicaid	1 [Reference]	1 [Reference]	1 [Reference]	1 [Reference]	1 [Reference]
Medicare	0.49 (0.16–1.50)	4.12 (0.98–17.38)	0.42 (0.13–1.33)	0.79 (0.16–4.00)	2.98 (0.74–11.93)
Private	**0.26 (0.11–0.64)**	**8.42 (2.42–29.33)**	1.14 (0.49–2.70)	0.57 (0.16–2.08)	0.96 (0.38–2.44)
Other	0.71 (0.12–4.3)	3.5 (0.44–28.14)	2 (0.31–13.06)	-	4.58 (0.46–45.61)

^3^Combines respondents who picked the survey options “I want to know exactly what I am taking and why” and “I want to understand the side effects of each medication thoroughly before accepting the prescription”.

Table 5 shows multivariate analysis of demographic predictors of the most frequent top choice for each question (Q1: “humanistic qualities”; Q2: “healthy lifestyle”; Q3: “SDM”; Q4: “SDM” and Q5: “knowing insurance coverage”). Those with younger age, higher educational attainment and private insurance had higher odds of ranking healthy lifestyle as a top choice. Those with more education had higher odds of ranking SDM as a top choice. Specifically, the lower odds of choosing “humanistic qualities” associated with private insurance compared to having Medicaid persisted here (0.21, CI 0.07–0.65) but higher education dropped out when controlling for all other demographic characteristics. In the personal responsibility domain, higher odds associated with private insurance and higher education (5.73, CI 1.36–24.27 and 2.98, CI 1.34–6.59 respectively) as well as the lower odds of older age choosing healthy lifestyle (0.23, CI 0.11–0.51 and 0.32, CI 0.10–0.97) persisted compared to their reference groups respectively. When controlled for other factors, having “Other” race dropped out as a significant predictor whereas being on Medicare appeared to have significantly higher odds of association with choosing a healthy lifestyle compared to the Medicaid insured, although still with a very wide Confidence Interval (5.98, CI 1.24–28.93). For tests and procedures, having college and above education remained associated with higher odds of choosing SDM (2.30, CI 1.06–4.99) compared to lower educational attainment. In the Medication category having “Other” race persisted as having higher odds of choosing SDM compared to Blacks (0.16, CI 0.04–0.61). The healthcare cost category remained without significant association with any of the demographics we tested in both uni and multi-variate analyses.

**Table 5:** Multivariate analysis for predictors of most frequent top choice for each question.

	Question #1 Patient-physician Relationship	Question #2 Personal Responsibility	Question #3 Tests and Procedures	Question #4 Medications	Question #5 Healthcare cost
Most frequent top choice	Humanistic Qualities	Healthy Lifestyle	Shared Decision Making (SDM)	Shard Decision Making^3^	Know Insurance coverage
	OR (95% C.I.)	OR (95% C.I.)	OR (95% C.I.)	OR (95% C.I.)	OR (95% C.I.)
Age (in years)					
< 35	1 [Reference]	1 [Reference]	1 [Reference]	1 [Reference]	1 [Reference]
35–44	0.84 (0.39–1.80)	0.49 (0.23–1.03)	1.47 (0.71–3.01)	0.33 (0.10–1.03)	1.24 (0.47–3.32)
45–64	0.61 (0.27–1.36)	**0.23 (0.11–0.51)**	1.24 (0.60–2.58)	0.27 (0.09–0.86)	0.74 (0.26–2.11)
> = 65	0.98 (0.33–2.93)	**0.32 (0.10–0.97)**	0.29 (0.08–1.01)	0.19 (0.04–0.1.02)	0.68 (0.17–2.66)
Sex					
Female	1 [Reference]	1 [Reference]	1 [Reference]	1 [Reference]	1 [Reference]
Male	0.72 (0.34–1.50)	0.86 (0.42–1.77)	0.57 (0.29–1.14)	0.64 (0.25–1.61)	0.58 (0.24–1.42)
Race					
Black	1 [Reference]	1 [Reference]	1 [Reference]	1 [Reference]	1 [Reference]
White	1.84 (0.82–4.12)	0.67 (0.32–1.44)	1.56 (0.75–3.23)	0.65 (0.21–2.01)	0.84 (0.30–2.32)
Other	1.49 (0.51–4.30)	0.81 (0.29–2.24)	1.45 (0.56–3.77)	**0.16 (0.04–0.61)**	0.46 (0.12–1.85)
Education					
Some college or	1 [Reference]	1 [Reference]	1 [Reference]	1 [Reference]	1 [Reference]
below		**2.98 (1.34–6.59)**	**2.30 (1.06–4.99**)	1.72 (0.58–5.12)	0.47 (0.16–1.37)
College and Above	0.66 (0.30–1.45)				
Insurance					
Medicaid	1 [Reference]	1 [Reference]	1 [Reference]	1 [Reference]	1 [Reference]
Medicare	0.42 (0.13–1.37)	**5.98 (1.24–28.93)**	0.46 (0.14–1.57)	0.96 (0.16–5.85)	3.36 (0.78–14.49)
Private	**0.21 (0.07–0.65)**	**5.73 (1.36–24.27)**	0.50 (0.17–1.48)	0.47 (0.09–2.53)	1.77 (0.53–5.90)
Other	0.81 (0.13–5.13)	4.74 (0.50–45.22)	2.97 (0.42–20.95)	-	6.71 (0.61–74.20)

^3^ Combines respondents who picked the survey options “I want to know exactly what I am taking and why” and “I want to understand the side effects of each medication thoroughly before accepting the prescription”.

## Discussion

Our study showed that in the patient-physician domain, humanistic quality was the most frequently ranked top 1–3 choice. This is consistent with other research findings which document that patient-physician interaction is viewed by most patients to be a highly important aspect of quality care.[10,17,25] The higher value our patient population seems to place on their physicians’ humanistic over clinical qualities such as the physician’s fund of knowledge could be explained by the fact that this survey was conducted in the ambulatory setting where a higher proportion of patients who may require significant emotional support are seen, an association that has been documented before.[3,19] Another possible reason why our patients showed a stronger preference for humanistic quality over clinical quality is that patients who come to reputable healthcare settings may assume that they will be cared for by practitioners with superior clinical abilities and hence tend to focus on their humanistic qualities instead. [52]

Although humanistic qualities appeared to be a highly valued choice for the domain of patient-physician relationship across the board, our uni and multi-variate analyses did show that the odds of choosing humanistic qualities was much lower for patients who had higher educational level (OR 0.55, CI 0.31–0.98) and or who were privately insured (OR 0.26, CI 0.11–0.64) as compared to lower educational level and Medicaid insured, a finding that has been noted before. [3,33] This may suggest that patients from lower socio-economic standing may have reasons to prioritize humanistic qualities in their care providers either because they don’t typically encounter this quality or because they may have increased needs for it due to their life circumstances.

In the personal responsibility domain, our findings of high correlation between prioritizing exercise, diet and leading a healthy lifestyle over other qualities with younger age, and higher educational attainment has been noted before. [39–42] This may be explained by the fact that, younger people are more agile, and a higher socio-economic standing (implied by higher educational attainment) may afford better access to healthy amenities as well as the fact that higher socio-economic standing may also confer the psychological space needed for people to prioritize healthy lifestyle over other concerns that may be at the top of their mind. [53–57] The higher odds of choosing healthy lifestyle seen in our multivariate analysis for those who have Medicare and Private insurance compared to the Medicaid insured (OR 5.98, CI 1.24–28.9 and OR 5.73, CI 1.36–24.27) is a significant finding and may once again be related to access to amenities in our patient population though this conclusion may carry less certainty for general interpretation due to the high confidence intervals.

SDM was the most frequently ranked top 1–3 choice for both “tests and procedures” and “medications” domains. The strong patient preference for SDM we found confirms the similar finding that has been noted before. [21,22] Clinicians will need to pay more attention to this aspect of care in the future as they will begin to see better informed patients come prepared to engage in decision making rather than to passively receive physician recommendations. The higher odds of choosing SDM by those with higher education in Q3 is also consistent with the evidence that better informed patients are likely to value and engage in SDM. [58,59] Explaining the higher odds we saw for choosing SDM for Q4 among those with “other” race would require a sub-subgroup analysis that was not performed here. In addition, loss of 53 surveys in this question may have reduced the power to detect other potentially significant associations in this category.

A question that asks patients to indicate their preference for knowing what their insurance covers and one that asks their preference to knowing what they are being charged for (two choices for Q5) is potentially confusing as one choice could be seen as a subset of the other. Despite that, it is clear that virtually all patients do care about the cost of care, especially the portion covered by insurance and/or themselves. Only a minority of those surveyed (11%) prioritized minimizing their healthcare expenditure which may indicate a related concept to the common health economics observation of moral hazard- where insured patients (virtually all our patients) may typically lack an incentive to prioritize healthcare cost minimization.

Our study has some weaknesses. The survey is liable to the inherent weakness of developing similar surveys discussed in the introduction. Patient preferences and priorities for various aspects of care are highly dynamic and complex and depend on personal, social, and other external factors including the health system where they receive their care. This has been seen in previously published data and in our own study here. For instance, a patient who may be highly anxious at a time of a serious diagnosis such as cancer may highly value the humanistic qualities of a physician but that same patient may value the physician’s clinical acumen at a later time once their acute psychological needs are met. For this reason, the validity of our survey in elucidating patients’ priorities for the domains tested may not always be accurate in different settings and under different conditions. We attempted to mitigate some of that by designing it using similar survey concepts published previously, [44] and piloting the survey before rolling out the project Our survey population was mostly privately insured females which may limit the generalizability of some of our findings, but we have demonstrated statistically significant results from our logistic regressions that is worth replicating in a larger study to evaluate these findings. Though our study showed differences in patient preferences along socio-economic status, preference for SDM on medications in the “other” race group was the only difference we observed by race. Prior studies show that differences in race and socio-economic status lead to differences in patients’ health care-related beliefs, practices, and values. [35–37] Prior research has also shown that race reflects multiple dimensions of social inequality and that indicators of SES capture aspects of racial differences. [60] We know in the United States, SES is strongly influenced by race. Our studies did show that Medicaid insured patients had higher odds of choosing the humanistic qualities of physicians than those respondents who were privately insured. Given most of our Medicaid insured patients are African Americans this may support the finding that SES does indeed capture race in this case.

Though we were able to get above a 90% response rate by providing self-administered questionnaire while patients were waiting to be seen in clinic, incomplete and inaccurate completion of surveys that were excluded may have caused selection bias in our patient samples in addition to reducing our power in the analysis of results. Several patients who erroneously completed the surveys ranked multiple choices equally. Although this may be due to our survey design needing more clarity (as in for Q5) one of the inherent difficulty of accurately capturing patient priorities is their unwillingness to trade between quality attributes, a finding seen in studies with discrete choice experiments. [3] Given the move towards a patient centered model of care delivery, it will be important for the future to develop a validated instrument that captures what matters to patients in different settings.

Our study contributes to the growing body of evidence that patient centeredness and understanding patient priorities are essential for value-based care. Our findings are in line with other published studies that suggest that humanistic qualities, [10,17,25] healthy lifestyle, [39–42] and SDM [21,22] are important. In addition, our results extend what is known by showing that patients still prioritize these qualities even when offered equally attractive alternatives, and these priorities are associated with certain patient level factors.

In conclusion, the delivery of effective and quality medical care requires understanding of what most matters to patients. The task of deciphering the multiple factors that may affect patient priorities for what they value is a real challenge and may be criticized for having biases related to wording and context. [16] However, it is still a useful endeavor that can help clarify further what we may be able to achieve in our move towards a VBC model that incorporates patients’ experience.

## Supporting information

S1 Appendix (XLSX)
